# Naloxone Use, 911 Calls, and Emergency Visits After Nonfatal Overdose

**DOI:** 10.1001/jamanetworkopen.2025.37678

**Published:** 2025-10-16

**Authors:** Brendan Saloner, Peter J. Fredericks, Lauren Byrne, Adrienne Hurst, Lindsey Kerins, Eric G. Hulsey, Julie Rwan, Sachini Bandara

**Affiliations:** 1 Department of Health Services, Policy, and Practice, Brown University School of Public Health, Providence, Rhode Island; 2 Department of Emergency Medicine, Johns Hopkins School of Medicine, Baltimore, Maryland; 3 Department of Health Policy and Management, Johns Hopkins Bloomberg School of Public Health, Baltimore, Maryland; 4 Vital Strategies, New York, New York; 5 Department of Mental Health, Johns Hopkins Bloomberg School of Public Health, Baltimore, Maryland

## Abstract

**Importance:**

People may not call 911 or visit the emergency department (ED) after a nonfatal overdose (NFOD), particularly when a layperson has already administered naloxone. Understanding service-engagement patterns can improve postoverdose care.

**Objective:**

To examine service use following an NFOD.

**Design, Setting, and Participants:**

This cross-sectional study of a telephone survey included adults who used cocaine, opioids, or methamphetamines in the prior 12 months. Respondents were recruited from harm-reduction, treatment, and social services organizations in New Jersey, Wisconsin, Michigan, and New Mexico from January 2023 to August 2024.

**Exposure:**

Experience with an NFOD in the prior 12 months.

**Main Outcomes and Measures:**

The main outcomes were use of naloxone, calls to 911, and ED visits following an NFOD and self-reported reasons for not calling 911. Adjusted odds ratios (AORs) were calculated to examine factors associated with each outcome and categorized free-response reasons if not engaging in care.

**Results:**

Among the 2097 participants (median [IQR] age, 42 years [34-52 years]; 1165 males [55.6%]), 538 (25.7%) had experienced at least 1 NFOD in the prior year. During the most recent NFOD, use of naloxone was reported by 430 of 524 survivors (82.1%), calls to 911 were reported by 328 of 535 survivors (61.3%), and visits to the ED were reported by 253 of 538 survivors (47.0%). In multivariable regressions of survivors of overdose, 911 was more likely to have been called at the most recent overdose event by people who resided in New Jersey (vs Wisconsin) (AOR, 3.24 [95% CI, 1.40-7.47]; *P* = .01), by non-Hispanic Black people (compared with non-Hispanic White people) (AOR, 1.79 [95% CI, 1.08-2.97]; *P* = .02), and by people who used drugs a few times a month at the time of the interview (vs no drug use) (AOR, 3.83 [95% CI, 1.23-12.00]; *P* = .02). The most commonly reported reasons for not calling 911 were that the person regained consciousness without naloxone (n = 61 [28.6%]) or that a bystander administered naloxone (n = 57 [26.8%]). Among those with a 911 call, visits to the ED were more common among Black individuals (AOR, 2.89 [95% CI, 1.11-7.54]; *P* = .03). Most people (n = 150 [61.5%]) received take-home naloxone in the hospital, and approximately one-fifth or fewer reported receiving buprenorphine (n = 57 [21.9%]) or methadone (n = 42 [16.2%]) before discharge.

**Conclusions and Relevance:**

In this cross-sectional study of survivors of drug overdose, fewer than half of recent overdose events did not culminate in a visit to the ED. These results suggest that policies to improve postoverdose outcomes must simultaneously focus on people who engage with emergency medical services and on those who do not seek formal medical care.

## Key Points

**Question:**

What services do people who use drugs commonly use following a nonfatal overdose, and what are common reasons for not engaging with care?

**Findings:**

In this cross-sectional study of a multistate telephone survey of 2097 people who use drugs, 538 had experienced a prior year overdose; of these, 61% had 911 called following a recent overdose, and 47% visited the hospital. Common reasons for not calling 911 included being able to regain consciousness without naloxone or having a bystander administer naloxone.

**Meaning:**

The findings of this study suggest that improving care for survivors of drug overdose may require improving the quality of emergency response services while also ensuring that lay responders have access to naloxone and overdose education.

## Teaser

This cross-sectional study of a multistate telephone survey of people who use drugs assesses responses regarding the services they use following a nonfatal overdose and common reasons for not engaging with care.

## Introduction

Drug overdose deaths in the US increased precipitously over the last decade, topping 100 000 deaths annually in 2021 and 2022. Since that peak, overdose deaths have decreased substantially, but the decrease has been slower among racial and ethnic minority populations, older adults, and in western states.^1^ In addition to reducing fatalities, increased efforts have been undertaken to improve treatment of nonfatal overdose (NFOD) events responded to by emergency medical services (EMS) and in hospital emergency departments (EDs). In the 12 months before March 16, 2025, federal data reported that EMS had about 650 000 encounters for an NFOD, and 17% resulted in patients not being transferred by medical personnel.^2^ Patients may decline transport to the ED for multiple reasons, including fear of mistreatment or arrest, acute discomfort and disorientation, and a lack of health insurance.^3^

Even before an EMS encounter, bystanders may not call 911, either because the patient believes that they can manage without medical treatment or because others in their social network opt to avoid medical services or law enforcement.^4,5^ The possibility of managing overdose without medical help has increased with greater availability of naloxone to people who use drugs (PWUD) and their social networks, which can be received through pharmacies or community-distribution programs. Even when naloxone is available, fear of precipitated withdrawal may lead to revival strategies that include rescue breathing without naloxone, sternum rubs, cold-water showers, or watchful waiting.^6^

To our knowledge, there are no survey-based estimates of the prevalence of medically untreated overdose events. The relatively limited data about service engagement in the aftermath of overdose events are primarily collected from relatively small or select groups.^3,4,7,8^ While these data have yielded important insights about some key reasons for care avoidance, much is still unknown about the experiences of this heterogeneous population. In the current study, we examined engagement with services after an NFOD drawing upon a large multistate survey of PWUD collected as part of a technical assistance campaign.

## Methods

### Survey Methods

In this cross-sectional study, we conducted a telephone-based survey (called VOICES) of PWUD (eAppendix in Supplement 1). Participants were recruited from harm-reduction, treatment, and social services organizations in 4 areas: Milwaukee, Wisconsin; Flint and Detroit, Michigan; statewide in New Jersey; and Bernalillo County, New Mexico, from January 2023 to August 2024. These sites are within states participating in the Bloomberg Overdose Prevention Initiative, a campaign that funds technical assistance and policy reform efforts. The study protocol was approved by institutional review boards at the Pacific Institute for Research and Evaluation and the Johns Hopkins Bloomberg School of Public Health, and participants provided oral informed consent. The study followed the Strengthening the Reporting of Observational Studies in Epidemiology (STROBE) reporting guideline.

Respondents were recruited from a total of 45 service organizations. Organizations distributed study recruitment cards that included a unique alphanumeric code and a toll-free hotline number. At some sites, respondents could share the code with up to 2 peers in their social network. Sites were selected to provide diversity in types of populations served, geographic coverage of the sampled areas, and racial and ethnic diversity. Methods have been described in a previous publication.^9^ The analytic sample is presented in the eFigure in Supplement 1.

Individuals calling the hotline were screened for eligibility, which included being at least 18 years of age, using opioids and/or stimulants (methamphetamine, crack, or cocaine) in the prior 12 months, and being able to consent and complete the survey. The hotline was staffed by trained interviewers, mostly during normal business hours in the time zone, and was offered in both English and Spanish. Surveys were designed to be completed in less than 30 minutes and covered topics such as drug use, perceptions of the drug supply, engagement with treatment and harm reduction, and structural vulnerabilities (eg, precarious housing). After completion, individuals were compensated with a $25 study gift card.

### Outcomes

All respondents were asked if they had experienced a drug overdose in the prior 12 months. If respondents were unsure, the interviewer would define an overdose as “a time when using drugs caused you to have serious problems breathing, you stopped breathing, you had an irregular or no heartbeat, you were unresponsive, or you required medical attention from a friend or an emergency responder.” Respondents endorsing an NFOD were asked the number of times. Respondents with at least 1 prior year NFOD were asked about whether the following events had occurred at the most recent overdose: (1) someone used naloxone, (2) someone called 911 for help, or (3) they were treated at the hospital ED. Respondents who said that 911 was not called were asked why not in a free-response question. If the respondent visited a hospital, they were asked if any of the following treatments were offered before discharge: methadone, buprenorphine, take-home naloxone, an appointment for drug treatment, or care for nonoverdose health issues (eg, wound care).

### Covariates

We included in our analysis measures associated with overdose risk and help-seeking behavior.^10,11,12^ These included demographics (age, sex, and race and ethnicity), recruitment state (Wisconsin, New Jersey, Michigan, and New Mexico), self-rated general health, structural vulnerabilities (recent food insecurity, criminal legal system involvement [defined as past-year time in jail or prison and/or current probation or parole], or worries about not having stable housing), current insurance status (dichotomized as Medicaid vs other non-Medicaid sources, such as uninsured or private insurance), substance use characteristics in the 30 days prior to the interview (types of substances used, frequency of use, and intentional mixing of substance types), number of prior-year overdoses (1 only, 2 or more), and current possession of naloxone. Race and ethnicity groups included Hispanic and the following non-Hispanic categories: Black, American Indian or Alaska Native, White, multiracial, other (including Asian American), and unknown. Race and ethnicity were ascertained by self-report and were included in this study because of the known differences in overdose mortality risk across racial and/or ethnic groups.

### Statistical Analysis

We calculated the crude prevalence of each of the outcome measures. We next calculated a series of logistic regression models that included the full set of covariates. With the full sample, we examined factors associated with experiencing 1 or more NFODs. Among those with 1 or more NFODs, we next examined factors associated with having naloxone used and having 911 called. Among those who had 911 called, we examined factors associated with going to the ED. SEs were clustered at the recruitment-site level to account for the sampling approach. We reported differences that were statistically significant at a 2-sided *P* < .05 level. Among participants who went to the ED, we also examined percentages receiving each service. We categorized free-response answers to the question, *Why not 911?* (including among respondents who went to the ED, since they may have not used 911 due to access to personal transportation to the hospital). We reported the frequencies of that measure. Data were analyzed using R statistical software, version 4.5.1 (R Project for Statistical Computing).

## Results

We collected 2183 participant responses, of which 2097 (median [IQR] age, 42 [34-52] years; 932 females [44.4%] and 1165 males [55.6%]) were included in our analytic sample. Overall, 538 people (25.7% of the analytic sample) experienced at least 1 NFOD in the prior year, and 314 of the NFOD sample (58.4%) had experienced 2 or more events (Table 1). The geographic distribution significantly differed between people with and without a prior-year NFOD. The most common state in the NFOD group was New Jersey with 169 of 538 respondents (31.4%) and in the no-overdose group, the most common state was New Mexico with 547 of 1559 respondents (35.1%) (eTable in Supplement 1). The median age of the NFOD group was 41 years (IQR, 33-52 years). Of the total participants, 571 (27.2%) were Black, 539 (25.7%) were Hispanic, 171 (8.1%) were American Indian or Alaska Native, 699 (33.3%) were White, 67 (3.2%) were multiracial, 40 (1.9%) were of other race or ethnicity, and 10 (<1.0%) were unknown.

**Table 1.** Descriptive Characteristics of Persons Experiencing Overdose Within the Prior 12 Months

Characteristic	No overdose (n = 1559)^a^	Overdose (n = 538)^a^	*P* value^b^
**Respondent**			
Recruitment state			
Wisconsin	356 (22.8)	122 (22.7)	<.001
New Jersey	403 (25.8)	165 (30.7)	
Michigan	267 (17.1)	133 (24.7)	
New Mexico	533 (34.2)	118 (21.9)	
Age, median (IQR), y	42 (34-53)	41 (33-52)	.08
Sex			
Female	702 (45.0)	230 (42.8)	.36
Male	857 (55.0)	308 (57.2)	
Race and ethnicity			
Hispanic	411 (26.4)	128 (23.8)	.09
Non-Hispanic			
American Indian or Alaska Native	135 (8.7)	36 (6.7)	
Black	434 (27.2)	137 (25.5)	
White	495 (31.8)	204 (37.9)	
Multiracial	48 (3.1)	19 (3.5)	
Other^c^	27 (1.7)	13 (2.4)	
Unknown	9 (0.6)	1 (0.2)	
Perceived health status			
Good, very good, or excellent	866 (55.5)	300 (55.8)	.93
Fair or poor	693 (44.5)	238 (44.2)	
Worried about stable housing	833 (53.4)	353 (65.6)	<.001
Difficulty affording household expenses	1138 (73.0)	442 (82.2)	<.001
Criminal legal involvement in past y	478 (30.7)	213 (39.6)	<.001
Insurance status			
Medicaid	1285 (82.4)	457 (84.9)	.29
Other	160 (10.3)	43 (8.0)	
Uninsured	114 (7.3)	38 (7.1)	
**Substance use **			
Current drugs used			
None	218 (14.0)	98 (18.2)	<.001
Opioids only	170 (10.9)	53 (9.9)	
Stimulants only	310 (19.9)	39 (7.2)	
Polysubstance use	861 (55.2)	348 (64.7)	
Frequency of drug use in past 30 d			
None	218 (14.0)	98 (18.2)	.004
Only once	32 (2.1)	9 (1.7)	
A few times/mo	142 (9.1)	40 (7.4)	
A few times/wk	343 (22.0)	87 (16.2)	
Once/d	129 (8.3)	35 (6.5)	
>Once/d	695 (44.6)	269 (50.0)	
Intentionally mixing drugs in the past 30 d	770 (49.4)	316 (58.7)	<.001
Currently possessing a naloxone kit	1058 (67.9)	426 (79.2)	<.001
No. of overdoses in past 12 mo			
Only 1	NA	224 (41.6)	NA
≥2	NA	314 (58.4)	NA
Outcomes for most recent overdose event^d^			
Bystander used naloxone	NA	430 (82.1)	NA
911 Called	NA	328 (61.3)	NA
Transported to emergency department	NA	253 (47.0)	NA

Abbreviation: NA, not applicable.

^a^ Data are presented as the No. (%) of respondents unless otherwise indicated.

^b^ Pearson χ^2^, Wilcoxon rank sum, and Fisher exact tests were used.

^c^ Includes Asian American.

^d^ Individuals with missing values for these measures were excluded in an analysis; thus for bystander used naloxone, n = 524; and for 911 called, n = 535.

Compared with people without an NFOD, people in the NFOD group were more likely to worry about housing stability (65.6% [353 of 538] vs 53.4% [833 of 1599]; *P* < .001), to have difficulty paying household expenses (82.2% [442 of 538] vs 73.0% [1138 of 1559]; *P* < .001), and to have criminal legal involvement (39.6% [213 of 538] vs 30.7% [478 of 1559]; *P* < .001). People who had a prior-year NFOD were more likely to be using opioids in combination with stimulants than those without an NFOD (64.7% [348 of 538] vs 55.2% [861 of 1559]; *P* < .001). People with a past-year NFOD were also more likely to report intentionally mixing drugs in the last 30 days than those who did not have an NFOD (58.7% [316 of 538] vs 49.4% [770 of 1559]; *P* < .001).

Among people with an NFOD, 430 of 524 (82.1%) reported that naloxone was used to treat their most recent overdose. About two-thirds (328 of 535 [61.3%]) of all respondents reported that 911 was called, and 253 of 538 respondents (47.0%) went to the ED after their most recent NFOD (Table 1).

Factors associated with 3 primary outcomes—naloxone being used by a bystander, 911 being called, and going to the ED for the most recent overdose event—among people with a prior-year NFOD are displayed in Table 2. The factors that were significantly associated with higher probability of naloxone being used were having had 2 or more overdoses in the past 12 months vs only 1 (adjusted odds ratio [AOR], 1.73 [95% CI, 1.02-2.96]; *P* = .04), mixing drugs in the last 30 days (AOR, 1.73 [95% CI, 1.00-2.96]; *P* = .048), and currently having naloxone in possession (AOR, 2.36 [95% CI, 1.44-3.86]; *P* < .001). Compared with people with Medicaid, being uninsured or having coverage from a source other than Medicaid was significantly associated with not having naloxone treat an overdose (AOR, 0.24 [95% CI, 0.13-0.48]; *P* < .01). Having 911 called was more likely to be reported among respondents in New Jersey compared with Wisconsin (AOR, 3.24; *P* = .006), and it was more frequent among Black individuals compared with White individuals (AOR, 1.79 [95% CI, 1.08-2.97]; *P* = .02) and among people who used drugs a few times a month vs none (AOR, 3.83 [95% CI, 1.23-12.00], *P* = .02). Conditional on having 911 called, the only factor that was significantly associated with use of the ED was Black race compared with White race (AOR, 2.89 [95% CI, 1.11-7.54]; *P* = .03).

**Table 2.** Characteristics Associated With Service Use at the Most Recent Nonfatal Overdose Event^a^

Characteristic	Model 1: bystander naloxone (n = 524)^b^		Model 2: 911 was called (n = 535)^c^		Model 3: transported to the emergency department (n = 328)^d^	
	Adjusted odds ratio (95% CI)	*P* value	Adjusted odds ratio (95% CI)	*P* value	Adjusted odds ratio (95% CI)	*P* value
Recruitment state						
Wisconsin	1 [Reference]	NA	1 [Reference]	NA	1 [Reference]	NA
New Jersey	1.30 (0.68-2.48)	.42	3.24 (1.40-7.47)	.006	1.80 (0.91-3.54)	.09
Michigan	1.69 (0.83-3.48)	.15	1.09 (0.50-2.38)	.84	1.55 (0.45-5.29)	.49
New Mexico	0.76 (0.41-1.42)	.38	0.59 (0.27-1.31)	.19	0.92 (0.45-1.87)	.82
Age, y	1.01 (0.99-1.03)	.43	1.01 (0.99-1.02)	.26	1.00 (0.97-1.02)	.69
Female sex	1.00 (0.57-1.75)	.99	1.29 (0.85-1.93)	.23	0.90 (0.51-1.61)	.73
Race and ethnicity						
Hispanic	0.70 (0.35-1.38)	.30	0.89 (0.52-1.52)	.68	0.66 (0.29-1.49)	.31
Non-Hispanic						
Black	0.88 (0.51-1.52)	.65	1.79 (1.08-2.97)	.02	2.89 (1.11-7.54)	.03
White	1 [Reference]	NA	1 [Reference]	NA	1 [Reference]	NA
Other^e^	0.76 (0.37-1.60)	.48	1.47 (0.73-2.96)	.28	1.06 (0.47-2.38)	.89
Perceived health status						
Good, very good, or excellent	1 [Reference]	NA	1 [Reference]	NA	1 [Reference]	NA
Fair or poor	0.92 (0.56-1.50)	.74	0.89 (0.66-1.21)	.48	1.43 (0.76-2.67)	.26
Worried about stable housing in next y	1.04 (0.66-1.66)	.85	1.26 (0.84-1.90)	.26	0.58 (0.27-1.24)	.16
Difficult to pay for basic needs in past y	0.73 (0.38-1.41)	.35	0.72 (0.43-1.22)	.23	1.38 (0.54-3.52)	.50
Criminal legal involvement in past y	1.38 (0.92-2.06)	.12	1.09 (0.72-1.67)	.68	1.29 (0.70-2.39)	.41
Insurance status						
Medicaid	1 [Reference]	NA	1 [Reference]	NA	1 [Reference]	NA
Other	0.24 (0.13-0.48)	<.001	1.02 (0.50-2.06)	.96	1.43 (0.52-3.96)	.49
Uninsured	1.07 (0.39-2.94)	.89	0.79 (0.29-2.18)	.65	1.40 (0.45-4.36)	.56
No. of overdoses in past 12 mo						
Only 1	1 [Reference]	NA	1 [Reference]	NA	1 [Reference]	NA
≥2	1.73 (1.02-2.96)	.04	1.09 (0.67-1.78)	.72	1.56 (0.90-2.70)	.12
Frequency of drug use in past 30 d						
None	1 [Reference]	NA	1 [Reference]	NA	1 [Reference]	NA
Only once	0.24 (0.04-1.49)	.13	1.33 (0.28-6.37)	.72	0.36 (0.02-5.11)	.45
A few times/mo	0.57 (0.19-1.74)	.33	3.83 (1.23-12.0)	.02	0.84 (0.24-3.02)	.79
A few times/wk	1.06 (0.44-2.59)	.89	1.45 (0.63-3.35)	.39	1.24 (0.44-3.48)	.68
Once/d	1.67 (0.40-6.96)	.47	1.50 (0.61-3.68)	.38	1.03 (0.28-3.72)	.97
>Once/d	0.70 (0.33-1.49)	.36	0.99 (0.45-2.16)	.98	1.08 (0.38-3.11)	.89
Drug mix in last 30 d	1.73 (1.00-2.96)	.048	0.66 (0.42-1.03)	.06	0.57 (0.27-1.17)	.13
Naloxone in possession	2.36 (1.44-3.86)	<.001	1.09 (0.60-1.99)	.78	0.59 (0.23-1.51)	.27

Abbreviation: NA, not applicable.

^a^ Multivariable logistic regression estimates. CIs and *P* values were adjusted for clustered sampling design.

^b^ All people who had at least 1 nonfatal overdose in the prior 12 months, aside from 11 with missing data (n = 524).

^c^ All persons with at least 1 nonfatal overdose (n = 535).

^d^ Respondents who had 911 called (n = 328).

^e^ Includes American Indian or Alaska Native and Asian American.

**Figure 1.**
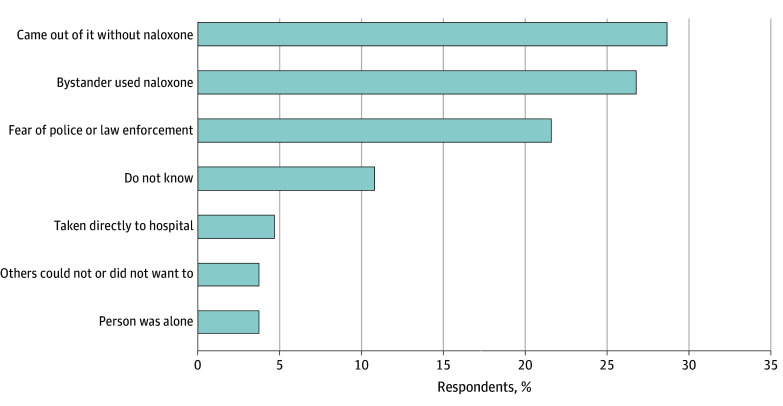
Self-Reported Reasons Why 911 Was Not Called After a Recent Nonfatal Overdose Analysis of free-response answers to the question of why 911 was not called following the most recent nonfatal overdose among a total of 213 respondents.

The most common self-reported reasons for 911 not being called are summarized in Figure 1. Among 213 respondents, the most commonly reported reason was that the person regained consciousness without naloxone (61 [28.6%]), the person came out of the overdose with bystander naloxone (57 [26.8%]), and fear of law enforcement (46 [21.6%]). Less commonly reported were factors such as being taken directly to the hospital without 911 being called (10 [4.7%]) and being alone (8 [3.8%]).

**Figure 2.**
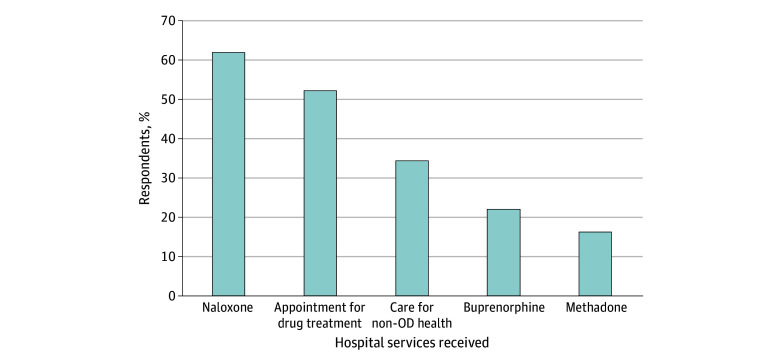
Services Received in the Hospital Following an Emergency Visit for a Nonfatal Overdose (OD) Analysis of 260 individuals who said that they went to the emergency department following their most recent nonfatal OD; individuals could endorse multiple options.

Frequencies for different services that 260 individuals received at the hospital are provided in Figure 2. The most commonly received service was take-home naloxone (150 [61.5%]) and an appointment for a drug-treatment program (135 [51.9%]). Less commonly reported was care for some nonoverdose-related health concern (89 [34.2%]) and buprenorphine (57 [21.9%]) or methadone (42 [16.2%]) administered during the hospital visit.

## Discussion

People who survive an NFOD have an elevated risk of subsequently dying of overdose,^13^ especially in the 2 days following the overdose event.^14^ Improving their care is critical to saving lives, yet there is limited public health guidance to support people not engaging with EMS. In this cross-sectional study of a multistate survey of people who experienced an NFOD in the prior year, we found that 61.3% of their most recent overdose experiences resulted in a 911 call and that roughly half (47.0%) were treated in a hospital. While there are not any current standard-of-care guidelines for treatment after an acute overdose,^15^ public health agencies advise the public to call 911 after an overdose and recommend that EMS and other first responders transport patients to the hospital for medical monitoring. However, our findings suggest that many people do not seek care via 911 calls and EMS.

People who did not call 911 commonly reported that they were able to manage without medical intervention. In some cases, this was because a bystander was able to revive the respondent with naloxone. In recent years, public health agencies have prioritized scaling up overdose education and naloxone distribution. Community access to naloxone has increased substantially in recent years, including through pharmacies, clinics, harm-reduction programs, and newer over-the-counter products.^16^ Naloxone distribution has also been supported through opioid settlement funds and federal grants and is an effective approach to reducing fatal outcomes.^17^ Better aligning bystander and peer-led programs with the formal care system could help to ensure that emergency services are available after naloxone is used.

Some people who did not use naloxone said that they did not use 911 because they came out of it in another way. It is unclear why in these cases naloxone was not used, but there is some qualitative literature indicating that PWUD often try other means to treat an overdose, such as rescue breathing, sternum rubs, or ice baths.^18^ People may try these approaches to avoid the precipitated withdrawal that accompanies naloxone use.^6^

Another reason why people either do not call 911 or do not agree to be transported to a hospital is the perception that they will not benefit, or could even be harmed, by engaging with services. Some respondents reported that 911 was not called because of potential negative repercussions, and others thought that medical services would not have been helpful. Prior experiences with stigma and discrimination from first responders and hospital staff may contribute to a desire to avoid medical care in the aftermath of an NFOD.^19^ Moreover, despite the legal protections under Good Samaritan laws, individuals may have justifiable concerns about being arrested, detained, or harassed by police or experiencing consequences associated with housing or child welfare if drug use is made visible.^20^

On the other hand, the ED can be a source of support and medical treatment. Notably, most people who were transported to the hospital received take-home naloxone and an appointment for drug treatment. Few respondents also received services from a peer and buprenorphine or methadone in the hospital. A lack of medications for opioid use disorder (MOUD) access during a hospital visit can contribute to greater patient discomfort from withdrawal and raise overdose risk and can also increase patient ambivalence about future engagements with medical services. Conversely, administering buprenorphine in the hospital has been associated with increased engagement with follow-up care.^21^ Furthermore, buprenorphine use immediately following a hospital visit can substantially reduce overdose risk in the following 6 months.^14^ Ensuring that MOUD access is widely available in hospitals is an important target for policies. States policies that encourage the adoption of MOUD in hospitals can have an important impact on patient outcomes after hospital visits for an NFOD, including greater take-up of buprenorphine.

Our study findings also indicate a role for interventions that do not depend on hospital-based care. For example, prehospital buprenorphine programs allow paramedics to administer buprenorphine on-scene to patients who have overdosed, rather than to wait for transport to the hospital. These programs have shown an association with improved continuing care and can benefit both patients who decline transport and those who receive further observation and care in the ED.^22,23^ A small number of communities have launched programs that engage with survivors of overdose using peer recovery workers or street-outreach teams, especially when individuals are reluctant to engage with EMS. Interventions with this model were operational in some of the communities that we surveyed, such as the Newark Community Street Team (Newark, New Jersey) and the Milwaukee Overdose Response Initiative (Milwaukee, Wisconsin), which partners the Fire Department with peer responders to assist with postoverdose outreach. These teams can encourage linkage to care but have largely not been yet evaluated for impact.^24^ Finally, stabilization centers (also known as sobering centers) provide a different destination for people who do not require complex medical intervention but can benefit from medical monitoring, linkage to care, and evaluation in a setting that is specifically designed to focus on people with a mental health or substance use crisis.^25^ Expanding these programs will require clear regulations (eg, permitting patients to be transported by first responders to a stabilization center in lieu of a hospital), reimbursement from public insurance programs, adequate staffing, and a clear mission to serve people in the aftermath of an overdose (beyond providing temporary sobering services).

Generally, we did not identify statistically significant sociodemographic or structural vulnerabilities associated with receipt of postoverdose care. One important exception was Black race compared with White race, which was significantly associated with higher adjusted odds of having 911 called and being transported to the ED. While Black PWUD are often more reluctant to engage with services due to higher levels of criminalization and stigma,^26,27^ another recent study using national EMS data also found that Black people are more likely than White people to accept transportation to the hospital after an overdose.^28^ Understanding potential racial and ethnic differences in postoverdose service use requires further investigation.

In addition to clinical practice, our study has important implications for public health surveillance. To our knowledge, this is the first large-scale survey of people who experienced an NFOD and provides the first estimate of what proportion of overdose events do not involve a call to 911. The findings extend and complement the existing literature using medical records and qualitative studies that have examined the decision to call 911 and to receive treatment in the hospital after an EMS encounter. Understanding how many overdose events occur without medical intervention is an important metric to help track acute health risks related to the unpredictable illicit drug supply and to understand how subgroups defined by geography, demographics, and drug-use behaviors are differentially experiencing these harms. Study data were collected during a time period in which fatal overdose rates in 3 of the 4 states (New Jersey, Wisconsin, and Michigan but not New Mexico) were declining overall from their historic height, although the trends were often not as favorable for minority populations.^29^ Tracking total NFODs that are not treated by EMS events can provide better insights into the effectiveness of broad-based community overdose prevention efforts.

### Limitations

This study has limitations. First, it relies on self-reports related to an NFOD, which is a stressful event associated with memory loss^30^ and therefore is subject to recall bias. Second, the questionnaire design denotes possession of naloxone and drug use around the time of the interview, rather than at the time of the overdose. It is therefore unclear whether these behaviors changed in response to the overdose event (eg, people who were abstinent at the time of the interview presumably had cut back drug use after their overdose event). Third, while this is a large and diverse community sample of people who have experienced an NFOD, it is limited to 4 geographic areas and primarily focuses on populations that were clients of drug-treatment or harm-reduction programs. The experiences of people who are disconnected from services may be different.

## Conclusions

In this cross-sectional study of people who experienced an NFOD, approximately half of all recent overdose events did not culminate in a visit to the hospital ED. This is likely to reflect both the ability to self-manage overdose and the perception that such services are not always beneficial and sometimes harmful. The lack of immediate service engagement could have longer-term implications for connection to treatment and harm reduction, which may influence subsequent risk of overdose. Engaging with survivors of overdose should be a critical national priority in an era of highly potent illicit drugs and requires improving care within the traditional EMS and hospital pathways and through nonclinical community response.

## Notes

Supplement 1. **eFigure.** Sample Selection Flowchart **eTable.** Descriptive Characteristics of Persons Experiencing Overdose Within the 12 Months by Recruitment State **eAppendix.** Survey

Supplement 2.
Data Sharing Statement
