# Supplementary material for: Naloxone Use, 911 Calls, and Emergency Visits After Nonfatal Overdose
Source: JAMA Netw Open. 2025 Oct 16;8(10):e2537678. doi: 10.1001/jamanetworkopen.2025.37678 (PMC12531882; doi:10.1001/jamanetworkopen.2025.37678)
Supplement: Supplement 1. — eFigure. Sample Selection Flowchart eTable. Descriptive Characteristics of Persons Experiencing Overdose Within the 12 Months by Recruitment State eAppendix. Survey [file jamanetwopen-e2537678-s001.pdf]

Supplemental Online Content

Saloner B, Fredericks PJ, Byrne L, et al. Naloxone use, 911 calls, and emergency visits after nonfatal overdose. *JAMA Netw Open*. 2025;8(10):e2537678. doi:10.1001/jamanetworkopen.2025.37678

eFigure. Sample Selection Flowchart
eTable. Descriptive Characteristics of Persons Experiencing Overdose Within the 12 Months by Recruitment State
eAppendix. Survey

This supplemental material has been provided by the authors to give readers additional information about their work.

© 2025 Saloner B et al. *JAMA Network Open*.

eFigure. Sample Selection Flowchart

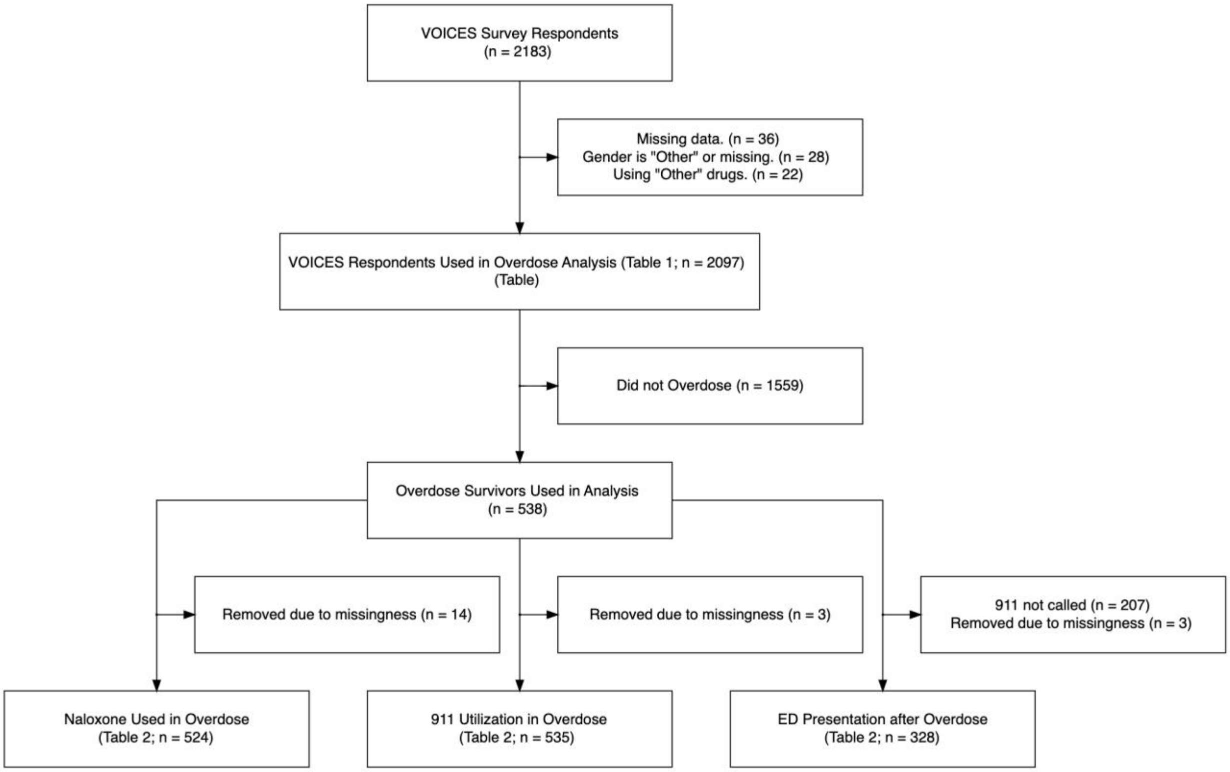

© 2025 Saloner B et al. *JAMA Network Open*.

eTable. Descriptive Characteristics of Persons Experiencing Overdose Within the 12 Months by Recruitment State

	**Wisconsin**, N = 498			**New Jersey**, N = 599			**Michigan**, N = 413			**New Mexico**, N = 673		
**Characteristic**	**No Overdose** N = 372[1]	**Overdose** N = 125[1]	**p-value**[2]	**No Overdose** N = 430[1]	**Overdose** N = 169[1]	**p-value**[2]	**No Overdose** N = 276[1]	**Overdose** N = 137[1]	**p-value**[2]	**No Overdose** N = 547[1]	**Overdose** N = 125[1]	**p-value**[2]
Age	47 (37, 56)	43 (35, 52)	0.030	44 (34, 55)	41 (33, 52)	0.15	41 (36, 52)	40 (32, 52)	0.2	38 (32, 46)	38 (31, 44)	0.6
Gender			0.3			>0.9			0.6			0.2
Male/Man	214 (58%)	63 (51%)		254 (60%)	100 (60%)		146 (53%)	79 (58%)		265 (49%)	69 (56%)	
Female/Woman	154 (42%)	61 (49%)		167 (39%)	67 (40%)		128 (47%)	57 (42%)		276 (51%)	53 (43%)	
Other	3 (0.8%)	0 (0%)		5 (1.2%)	1 (0.6%)		1 (0.4%)	0 (0%)		5 (0.9%)	2 (1.6%)	
Race/Ethnicity			0.12			0.12			0.3			0.7
NH Black	162 (44%)	46 (37%)		194 (46%)	59 (35%)		76 (28%)	32 (24%)		15 (2.8%)	2 (1.6%)	
Non-Hispanic (NH) White	125 (34%)	50 (40%)		124 (29%)	62 (37%)		161 (59%)	73 (54%)		111 (20%)	25 (20%)	
Hispanic	48 (13%)	19 (15%)		86 (20%)	38 (22%)		17 (6.2%)	16 (12%)		269 (49%)	63 (50%)	
NH Multirace	13 (3.6%)	7 (5.6%)		8 (1.9%)	5 (3.0%)		11 (4.0%)	7 (5.2%)		18 (3.3%)	1 (0.8%)	
NH Native	12 (3.3%)	0 (0%)		2 (0.5%)	0 (0%)		2 (0.7%)	2 (1.5%)		125 (23%)	34 (27%)	
NH Other	6 (1.6%)	3 (2.4%)		9 (2.1%)	4 (2.4%)		7 (2.6%)	5 (3.7%)		4 (0.7%)	0 (0%)	
NH Asian	0 (0%)	0 (0%)		0 (0%)	1 (0.6%)		0 (0%)	0 (0%)		3 (0.6%)	0 (0%)	
Perceived Health Status			0.8			0.12			0.8			0.2
Good/Very good/Excellent	194 (52%)	67 (54%)		216 (51%)	98 (58%)		167 (61%)	80 (59%)		313 (57%)	63 (51%)	
Fair/Poor	176 (48%)	57 (46%)		206 (49%)	70 (42%)		109 (39%)	55 (41%)		232 (43%)	61 (49%)	
Worried about Stable Housing	170 (46%)	72 (58%)	0.019	236 (56%)	112 (67%)	0.013	122 (44%)	81 (60%)	0.003	340 (62%)	97 (78%)	<0.001

© 2025 Saloner B et al. *JAMA Network Open*.

	**Wisconsin**, N = 498			**New Jersey**, N = 599			**Michigan**, N = 413			**New Mexico**, N = 673		
**Characteristic**	**No Overdose** N = 372[1]	**Overdose** N = 125[1]	**p-value**[2]	**No Overdose** N = 430[1]	**Overdose** N = 169[1]	**p-value**[2]	**No Overdose** N = 276[1]	**Overdose** N = 137[1]	**p-value**[2]	**No Overdose** N = 547[1]	**Overdose** N = 125[1]	**p-value**[2]
Difficulty Affording Household Expenses	247 (67%)	93 (74%)	0.11	314 (74%)	143 (85%)	0.004	188 (68%)	114 (84%)	<0.001	430 (79%)	108 (86%)	0.049
Criminolegal Involvement	73 (20%)	52 (42%)	<0.001	107 (25%)	51 (30%)	0.2	94 (34%)	56 (41%)	0.2	213 (39%)	61 (49%)	0.043
Insurance Status			0.4			0.072			0.071			0.8
Medicaid	292 (78%)	104 (83%)		312 (73%)	137 (81%)		240 (87%)	114 (83%)		482 (88%)	113 (90%)	
Other	62 (17%)	18 (14%)		54 (13%)	12 (7.1%)		29 (11%)	13 (9.5%)		35 (6.4%)	6 (4.8%)	
Uninsured	18 (4.8%)	3 (2.4%)		64 (15%)	20 (12%)		7 (2.5%)	10 (7.3%)		30 (5.5%)	6 (4.8%)	
**Substance Use Characteristics**												
Current Drugs Used			<0.001			0.004			<0.001			<0.001
None	66 (18%)	33 (26%)		63 (15%)	29 (17%)		45 (16%)	19 (14%)		48 (8.8%)	18 (14%)	
Opioids Only	37 (9.9%)	15 (12%)		77 (18%)	22 (13%)		27 (9.8%)	13 (9.5%)		35 (6.4%)	4 (3.2%)	
Stimulants Only	101 (27%)	11 (8.8%)		65 (15%)	12 (7.1%)		75 (27%)	15 (11%)		83 (15%)	4 (3.2%)	
Polysubstance + Stimulant Use	166 (45%)	66 (53%)		217 (50%)	106 (63%)		125 (45%)	89 (65%)		375 (69%)	97 (78%)	
Other	2 (0.5%)	0 (0%)		8 (1.9%)	0 (0%)		4 (1.4%)	1 (0.7%)		6 (1.1%)	2 (1.6%)	
Frequency of Drug Use in past 30 days			0.019			0.5			0.5			0.034
None	66 (18%)	33 (26%)		63 (15%)	29 (17%)		45 (16%)	19 (14%)		50 (9.1%)	20 (16%)	
Only once	7 (1.9%)	0 (0%)		10 (2.3%)	4 (2.4%)		8 (2.9%)	4 (2.9%)		9 (1.6%)	2 (1.6%)	
A few times a month	52 (14%)	15 (12%)		48 (11%)	14 (8.3%)		18 (6.5%)	8 (5.8%)		40 (7.3%)	4 (3.2%)	
A few times a week	104 (28%)	23 (18%)		125 (29%)	42 (25%)		45 (16%)	15 (11%)		85 (16%)	10 (8.0%)	

© 2025 Saloner B et al. *JAMA Network Open*.

	Wisconsin, N = 498			New Jersey, N = 599			Michigan, N = 413			New Mexico, N = 673		
**Characteristic**	**No Overdose** N = 372[1]	**Overdose** N = 125[1]	**p-value**[2]	**No Overdose** N = 430[1]	**Overdose** N = 169[1]	**p-value**[2]	**No Overdose** N = 276[1]	**Overdose** N = 137[1]	**p-value**[2]	**No Overdose** N = 547[1]	**Overdose** N = 125[1]	**p-value**[2]
Once a day	33 (8.9%)	6 (4.8%)		39 (9.1%)	12 (7.1%)		27 (9.8%)	11 (8.0%)		37 (6.8%)	9 (7.2%)	
More than once a day	110 (30%)	48 (38%)		145 (34%)	68 (40%)		133 (48%)	80 (58%)		326 (60%)	80 (64%)	
Intentionally Mixing Drugs in the past 30 days	157 (42%)	58 (46%)	0.4	201 (47%)	95 (56%)	0.037	122 (44%)	89 (65%)	<0.001	318 (58%)	83 (66%)	0.093
Naloxone kit	197 (53%)	88 (70%)	<0.001	288 (67%)	131 (78%)	0.013	203 (74%)	122 (89%)	<0.001	413 (76%)	99 (79%)	0.4
Number of overdoses in past 12 Months			>0.9			>0.9			>0.9			>0.9
Only 1	0 (NA%)	49 (39%)		0 (NA%)	78 (46%)		0 (NA%)	51 (37%)		0 (NA%)	57 (46%)	
2 or more	0 (NA%)	76 (61%)		0 (NA%)	91 (54%)		0 (NA%)	86 (63%)		0 (NA%)	68 (54%)	
Someone Used Naloxone Most Recent Overdose	0 (NA%)	97 (80%)	>0.9	0 (NA%)	137 (84%)	>0.9	0 (NA%)	119 (89%)	>0.9	0 (NA%)	92 (75%)	>0.9
911 Called	0 (NA%)	75 (60%)	>0.9	0 (NA%)	136 (80%)	>0.9	0 (NA%)	77 (57%)	>0.9	0 (NA%)	50 (41%)	>0.9
Presented to Emergency Department	0 (NA%)	58 (46%)	>0.9	0 (NA%)	112 (66%)	>0.9	0 (NA%)	61 (45%)	>0.9	0 (NA%)	29 (23%)	>0.9

[1]Median (Q1, Q3); n (%)

[2]Wilcoxon rank sum test; Fisher's exact test; Pearson's Chi-squared test

© 2025 Saloner B et al. *JAMA Network Open*.

Survey

Record ID	__________________________
**I'm going to begin recording now. It will take just a few moments to do that.<br><br>Start recording.<br><br>Once recording has started, make a verbal note that will be at the beginning of the recording to track which record ID the recording goes with: "This is record ID _______" [state record ID which can be found at top of survey form]**	
Now I'm going to ask you questions about the past month, so please think back from _____ through today when answering.<br><br>I'm going to read a list of drug types. Please tell me if you have used each drug in the past month by saying yes or no.<br><br>Do not read. Other examples of types of prescription drug may include:<br><br>Opioid pills: Percodan, percocet<br><br>Tranquilizers: Valium<br><br>Stimulants: Preludin	☐ Cocaine or crack<br>☐ Heroin<br>☐ Fentanyl<br>☐ Crystal methamphetamine, speed, or other methamphetamine<br>☐ Opioid pain pills such as Oxycontin, not prescribed to you<br>☐ Tranquilizers or antianxiety drugs such as benzos or Xanax, not prescribed to you<br>☐ Stimulants such as Ritalin or Adderall, not prescribed to you<br>☐ Buprenorphine, Suboxone, methadone not prescribed to you<br>☐ (do not read - select if no to all of above) Did not use any of these drugs in the past month
In the past month, did you intentionally mix or combine drugs and use them at the same time?	◯ Yes<br>◯ No
Which drugs did you intentionally use at the same time?<br><br>Do not read. Check all that apply based on response.	☐ Heroin and fentanyl together<br>☐ Cocaine/crack and heroin/opioid together (speedball)<br>☐ Crystal methamphetamine and heroin/opioid together (goofball)<br>☐ Other
Please specify the other types of drugs used at the same time.	__________________________
In the past month, how often did you use drugs (was it... a few times a week, a few times a month, or more often)?<br><br>Do not read. Pick choice that best applies based on response. Prompt with response options if needed.	◯ More than once a day<br>◯ Once a day<br>◯ A few times a week<br>◯ A few times a month<br>◯ Only once

© 2025 Saloner B et al. *JAMA Network Open.*

In the past month, which ways have you taken drugs (like injecting, smoking, etc.)?<br><br>Let respondent answer, check all that apply and confirm that any modes of drug use not endorsed were not used.	☐ Injected<br>☐ Smoked<br>☐ Snorted/sniffed<br>☐ Swallowed<br>☐ Some other way
Please specify the other way(s) drugs taken.	__________________________

Overdose Experience

Now I'm going to ask you some questions about your experience with overdose.

Now think back to the last 12 months. Have you had a drug overdose in the last 12 months, that is since __________.<br><br>Read prompt below if needed.<br><br>For the purposes of this survey, an overdose is defined as a time when using drugs caused you to have serious problems breathing, you stopped breathing, you had irregular or no heartbeat, you were unresponsive, or you required medical attention from a friend, bystander, or emergency responder.	◯ Yes<br>◯ No
In the last year, how many drug overdoses have you had?	__________________________

For the next set of questions, think back to the most recent time you had an overdose.

At the time of your most recent overdose, did someone use naloxone (also called Narcan) on you to reverse the overdose?	◯ Yes<br>◯ No<br>◯ Don't know
Did someone call 911 for help?	◯ Yes<br>◯ No<br>◯ Don't know
Any specific reason why not?	__________________________
Were you treated at a hospital emergency department?	◯ Yes<br>◯ No
Any specific reason why not?	__________________________

Before you left the hospital, were you offered any of the following?<br><br>I'll read the list and you can say yes/no.	☐ Methadone<br>☐ Buprenorphine<br>☐ Naloxone or Narcan that you can take home<br>☐ Appointment for a drug treatment program<br>☐ Care for other non-overdose health issues (wound care or other health needs)
Before you left the hospital, were you offered any of the following?<br><br>I'll read the list and you can say yes/no.	☐ Methadone<br>☐ Buprenorphine<br>☐ Naloxone or Narcan that you can take home<br>☐ Appointment for a drug treatment program<br>☐ Care for other non-overdose health issues (wound care or other health needs)<br>☐ A visit from a peer recovery worker

Overdose Risk Perception

I know you told me that you did not use any drugs in the last month, but I am still going to ask you a few questions about your perceptions of future risk.

How worried are you about using drugs that are mixed or cut with substances that you did not intend to use? Are you...<br><br>Read response list	◯ Very worried<br>◯ Quite a bit worried<br>◯ Just a little bit worried<br>◯ Not at all worried
How worried are you about having an overdose in the future? Are you...<br><br>Read response list.	◯ Very worried<br>◯ Quite a bit worried<br>◯ Just a little bit worried<br>◯ Not at all worried

Harm Reduction Services Now I'm going to ask some questions about your use of harm reduction services.

Harm reduction services include safer drug use supplies like naloxone/Narcan, fentanyl test strips, sterile needles, and safer smoking equipment.

In the past month, have you received any harm reduction services?	◯ Yes<br>◯ No
Any specific reason why not?<br><br>Do not read the list. Select all applicable responses, and prompt other responses from list if needed.	☐ You feel you don't need harm reduction services<br>☐ You don't know about harm reduction services in your area<br>☐ You don't have transportation to get there<br>☐ The hours don't work with your schedule<br>☐ You're worried people might think poorly of you<br>☐ You're worried program staff will treat you poorly<br>☐ You're worried about getting arrested by the police after going<br>☐ Some other reason
Please specify the other reason.	__________________________

© 2025 Saloner B et al. *JAMA Network Open.*

In the past month, what harm reduction supplies or services did you receive?<br><br>Do not read. Check off based on responses and then clarify any remaining supplies not mentioned.<br><br>If respondent says doesn't remember, ask "If you can't remember what you got within the past 30 days, what sort of supplies do you usually receive from the harm reduction program?"	☐ New needles or other safer injection supplies (e.g. cooker, water, cottons, etc.)<br>☐ Smoking supplies<br>☐ Snorting supplies<br>☐ Naloxone or Narcan<br>☐ Fentanyl test strips<br>☐ Wound care supplies<br>☐ An HIV, Hep C, TB, or syphilis test<br>☐ Other<br>☐ None of the above
Please specify other supplies or services received.	__________________________
Overall, in the past month, how would you rate your experience receiving harm reduction services? Was it...<br><br>Read list	○ Very good<br>○ Good<br>○ Acceptable<br>○ Poor<br>○ Very poor
What, if anything, would improve your experience receiving harm reduction services?	________________________________
Are there any drug use supplies that you want that you cannot currently get?	__________________________

Drug Testing Tools

Now I want to ask about fentanyl test strips which can be used to quickly check if the drugs contain fentanyl.<br><br>Have you used fentanyl test strips in the past month?	○ Yes<br>○ No
Any specific reason why not?<br><br>Do not read list. Select all responses that best fit answer.	☐ I do not want to<br>☐ I do not know about test strips<br>☐ I do not have any test strips<br>☐ It is too difficult to get test strips<br>☐ Testing takes too long<br>☐ I don't mind using fentanyl<br>☐ I am worried about what people might think if I use test strips<br>☐ I am worried about getting arrested for having fentanyl test strips<br>☐ I do not know where to get test strips<br>☐ Other
Please enter the other reason(s) for not using test strips.	________________________________
The last time you used fentanyl test strips, did your drugs test positive for fentanyl?	○ Yes<br>○ No

What did you do when your drugs tested positive? Did you...<br><br>Read list. Note that this refers to the last time their drugs tested positive for fentanyl.	☐ Continue using as usual<br>☐ Use less<br>☐ Use more slowly<br>☐ Not use the drugs at all<br>☐ Use with someone else or have someone check on you
What, if anything, would encourage you to use fentanyl test strips more regularly?	____

Naloxone Possession

Do you have a naloxone kit?<br><br>If person is confused mention that naloxone is sometimes called Narcan.	◯ Yes<br>◯ No
Where did you get your naloxone/Narcan kits?<br><br>Do not read list. Select all responses that best fit answer.	☐ Pharmacy<br>☐ Doctor's office<br>☐ Harm reduction organization<br>☐ Drug treatment program<br>☐ A family member or friend<br>☐ Prison/jail<br>☐ Vending machine<br>☐ A mail order program<br>☐ NaloxBox - a small box in public locations that has overdose response supplies<br>☐ Other
Please specify other places where got naloxone kits.	________________________________
If somebody overdoses while you are with them, how comfortable are you with using naloxone/Narcan on them? Are you...<br><br>Read list	◯ Completely comfortable<br>◯ Fairly comfortable<br>◯ Slightly comfortable<br>◯ Not comfortable at all
What, if anything, would increase your confidence to use naloxone/Narcan to reverse an overdose?	____

Substance Use Disorder Treatment Services

Now I'd like to ask you some questions about experiences you've had with drug treatment services, if any.

In the past month have you attended a group like Narcotics Anonymous, SMART recovery or other peer-led group?	◯ Yes<br>◯ No
Separate from groups, have you received treatment, medication, or counseling for your use of drugs from a medical provider or professional counselor in the past month?	◯ Yes<br>◯ No

© 2025 Saloner B et al. *JAMA Network Open*.

Have you wanted to receive treatment, medication, or counseling for your use of drugs in the past month?	◯ Yes<br>◯ No
In the last month, at what kinds of places have you received treatment, medication, or counseling for drug use? It can be more than one.<br><br>Do not read list. Select all applicable responses, and prompt other responses from list if needed.	☐ Doctor's office, or community health center<br>☐ Methadone Clinic<br>☐ Emergency room<br>☐ Hospital overnight<br>☐ Residential drug rehab facility<br>☐ Drug rehab facility as an outpatient<br>☐ Prison or jail<br>☐ Mobile treatment van<br>☐ Sober living facility/recovery housing<br>☐ Other
Please specify the other types of places treatment received.	______________________________
I am going to read a list of medications you might have received as treatment for drug use. For each one, please tell me if you received this medication in the past month, if at all.<br><br>Read each of the first three options and check if "yes". Only check "Did not receive any of these medications" if nothing on the list is endorsed by the participant.	☐ Buprenorphine or Suboxone<br>☐ Methadone<br>☐ Naltrexone/Vivitrol<br>☐ (Don't read) Did not receive any of these medications
Have you been given permission to take home your methadone?	◯ Yes<br>◯ No
How many days of supply do you get to take home?	______________________________
I am going to read a list of experiences you may have had at a pharmacy when you pick up your buprenorphine or Suboxone. Please tell me whether you experienced this in the past month by saying yes or no.	☐ The pharmacy did not have buprenorphine in stock<br>☐ The pharmacy staff treated you poorly<br>☐ The pharmacy staff gave you helpful information about how to take buprenorphine
In a sentence or two, please tell me your goals for treatment.<br><br>Prompts if respondent needs clarification: Were there any reasons you wanted to start treatment? How do you think or hope your life may change as a result of being in treatment? What do you hope will happen by going to treatment?	______________________________________
In the past month, how much did your treatment for drug use help you reach your goals?<br><br>Did it help....<br><br>Read list	◯ Not at all<br>◯ A little<br>◯ A lot

Question	Response
In the past month, how respectful were the treatment staff towards you?<br><br>Read list<br><br>Prompt, if needed, "on average, thinking about all of your experiences in the past 30 days, how respectful were the treatment staff towards you?"	◯ Not at all<br>◯ A little<br>◯ A lot
There are many reasons why people may not get treatment even if they want it. Has this ever happened to you?	◯ Yes<br>◯ No
What are the reasons why you have not gotten treatment even when you wanted to.<br><br>Do not read list. Select all that best fit response.	☐ Cost<br>☐ No insurance<br>☐ Concerned about being treated poorly<br>☐ Inconvenient times<br>☐ Inconvenient locations<br>☐ Transportation<br>☐ Childcare<br>☐ Too many rules<br>☐ Was in jail or prison<br>☐ The program could not accommodate your other healthcare or disability needs<br>☐ Concerned about what other people will think/embarrassed<br>☐ Not ready<br>☐ Could not find a provider who speaks preferred language or provides an interpreter when needed<br>☐ Other<br>☐ Did not experience any barriers
Please specify other challenge(s)	__________________________
I'm going to read you a statement. Please tell me how strongly you agree or disagree with this statement.<br><br>It is more effective to treat opioid addiction with medications like methadone, buprenorphine, or naltrexone than to treat opioid addiction without medication. Would you say that you...<br><br>Read list	◯ Strongly agree<br>◯ Agree<br>◯ Disagree<br>◯ Strongly disagree

Structural Vulnerability

Now I am going to ask you some questions about your living situation and other life experiences.

Question	Response
What is your current employment status?<br><br>Do not read list. Check answer that best fits and use list as prompt if needed.	◯ Employed full time<br>◯ Employed part time<br>◯ Retired<br>◯ Unemployed due to health reason<br>◯ Unemployed due to non-health reason<br>◯ Other

© 2025 Saloner B et al. *JAMA Network Open*.

In what zip code do you live or spend most of your time?<br><br>If they cannot remember zip code then ask: Can you tell us a major intersection or landmark in that area?	__________________________
Are you worried or concerned that in the next year you may not have stable housing that you own, rent, or stay in as a part of a household?	◯ Yes<br>◯ No
In the past year, has it been difficult for you to pay for your basic needs, like food, housing, or other bills?	◯ Yes<br>◯ No
Are you currently responsible for any children under the age of 18?<br><br>Do not read unless clarification needed: This can refer to children in your legal custody or those living in your household who you care for that are not in your legal custody.	◯ Yes<br>◯ No
In the past year, have you been involved with child protective services regarding a child that is under your care?	◯ Yes<br>◯ No
In the past year have you spent any time in prison or jail?	◯ Yes<br>◯ No<br>◯ Prefer not to answer
In the past year have you been on probation, parole, supervised release or other conditional release from jail or prison?	◯ Yes<br>◯ No<br>◯ Prefer not to answer
In the past year, have you been unfairly stopped, questioned, or searched by the police?	◯ Yes<br>◯ No
In the past year have you been arrested for possession of drugs or drug-related paraphernalia?	◯ Yes<br>◯ No
Where were you at the time of your most recent arrest?<br><br>Do not read. Select choice that best applies based on response. Prompt with response options if needed.	◯ In a private residence, including your own home<br>◯ In your car<br>◯ On a bus, or other public transportation<br>◯ In a public place<br>◯ In a hospital or health care facility<br>◯ At a harm reduction or syringe access program<br>◯ At a treatment program<br>◯ At some other place
Please specify the place the arrest was made.	__________________________
What was the reason for your most recent drug or drug-related arrest?	__________________________________

© 2025 Saloner B et al. *JAMA Network Open*.

Have you ever felt unfairly judged or discriminated against based on your race, ethnicity, gender, sexual orientation or a health condition?	◯ Yes<br>◯ No
Have you ever been made to feel this way by the following people?<br><br>Read list and check all that apply.	☐ Drug treatment providers<br>☐ Harm reduction staff<br>☐ Police<br>☐ EMS, fire department, or paramedics<br>☐ Emergency Department<br>☐ Other medical providers<br>☐ Someone else
Please specify who has made you feel this way.	______________________

Socio-demographic Characteristics   We just have a few more questions left.

Are you of Hispanic, Latino, or Spanish origin?	◯ Yes<br>◯ No
What is your race?<br><br>Select all that apply	☐ Black or African American<br>☐ White<br>☐ Asian<br>☐ American Indian, Native American or Alaska Native<br>☐ Something else
Please specify race	______________________
What is your gender?	◯ Female/Woman<br>◯ Male/Man<br>◯ Non-binary<br>◯ Another gender category
Please specify gender	______________________
Do you consider yourself to be transgender?	◯ Yes<br>◯ No
What is your sexual orientation?	◯ Gay, lesbian, or homosexual<br>◯ Heterosexual or straight<br>◯ Bisexual<br>◯ Something else
Please specify your sexual orientation	______________________

© 2025 Saloner B et al. *JAMA Network Open.*

Who do you currently live with? If you live alone, please let me know.<br><br>Select all that apply. Choose answer(s) that fits best. Do not read list unless needed as prompt.	☐ Alone or by yourself<br>☐ A spouse<br>☐ A non-spouse partner<br>☐ Your parent or parents<br>☐ Your child or children under 18<br>☐ Your adult child or children<br>☐ Your grandparent or grandparents<br>☐ Your grandchild or grandchildren<br>☐ Other family members<br>☐ A roommate who is not a partner or family member<br>☐ Lives with other people at a drug treatment program or recovery housing<br>☐ Other
Please specify who they live with	__________________________

General Health/Wellness

Would you say your health in general is excellent, very good, good, fair or poor?	◯ Excellent<br>◯ Very good<br>◯ Good<br>◯ Fair<br>◯ Poor
Has a doctor or other health professional ever told you that you have a chronic or serious medical condition?<br><br>If yes, read: Please tell me which ones.<br><br>Select all that apply.<br><br>If no, check "No chronic or serious medical conditions"	☐ A heart condition<br>☐ Diabetes<br>☐ High blood pressure<br>☐ Cirrhosis of the liver<br>☐ Hepatitis B or C<br>☐ HIV or AIDS<br>☐ Kidney disease<br>☐ Cancer<br>☐ Chronic bronchitis or chronic obstructive pulmonary disease (COPD)<br>☐ Depression or anxiety<br>☐ Bipolar disorder or schizophrenia<br>☐ Chronic pain<br>☐ Arthritis<br>☐ Asthma<br>☐ Other<br>☐ No chronic or serious medical conditions<br>☐ Do not know
Please specify other chronic or serious health condition	__________________________

Do you have serious difficulty concentrating, remembering, or making decisions because of a physical, mental, or emotional condition?<br><br>If participant responds "sometimes", then check "yes".<br><br>Add clarification if needed that we are asking about these symptoms being caused by a "physical, mental, or emotional condition".	◯ Yes<br>◯ No
Do you have serious difficulty walking or climbing stairs?	◯ Yes<br>◯ No
What type of health insurance or health coverage plan do you currently have, if any?<br><br>Pick the response that best fits. Use list as prompt if necessary.<br><br>Select all that apply	☐ Private insurance received through an employer, union or purchased directly through an insurance company (either through yourself or a family member)<br>☐ Medicare for people 65 and older or people with certain disabilities<br>☐ Medicaid, Medical Assistance, BadgerCare (WI), NJ Family Care (NJ), Healthy Michigan Plan (MI), or any kind of government-assistance plan for those with low incomes or disabilities<br>☐ TRICARE, VA or other military or veteran health care<br>☐ Indian Health Service<br>☐ Uninsured, no insurance<br>☐ Other
We've been asking you a lot of questions about your day to day life. In a few sentences, can you please tell me what your future health and wellness goals are?<br><br>Prompts if needed: this may include your physical health, mental health, spiritual health or changes related to your job or housing, your relationships, or your overall quality of life.	__________________________________
What would support you in achieving your goals?	__________________________________
Is there anything else that we did not discuss that you would like to share?	__________________________________
We have reached the end of the survey. I am now going to stop the recording.<br><br>Stop the recording.	
Please add any notes about this record here.	__________________________________
