# Supplementary material for: Naloxone Use, 911 Calls, and Emergency Visits After Nonfatal Overdose
Source: JAMA Netw Open. 2025 Oct 16;8(10):e2537678. doi: 10.1001/jamanetworkopen.2025.37678 (PMC12531882; doi:10.1001/jamanetworkopen.2025.37678)
Supplement: Supplement 2. — Data Sharing Statement [file jamanetwopen-e2537678-s002.pdf]

Data Sharing Statement

Saloner. Naloxone Use, 911 Calls, and Emergency Visits After Nonfatal Overdose. *JAMA Netw Open*. Published October 16, 2025. doi:10.1001/jamanetworkopen.2025.37678

Data
Data available: No

Additional Information
Explanation for why data not available: Participants were not consented for data sharing
